# Systematic review and meta-analysis of randomized clinical trials comparing efficacy and safety outcomes of insulin glargine with NPH insulin, premixed insulin preparations or with insulin detemir in type 2 diabetes mellitus

**DOI:** 10.1007/s00592-014-0698-4

**Published:** 2015-01-14

**Authors:** Przemyslaw Rys, Piotr Wojciechowski, Agnieszka Rogoz-Sitek, Grzegorz Niesyczyński, Joanna Lis, Albert Syta, Maciej T. Malecki

**Affiliations:** 1 HTA Consulting, Kraków, Poland; 2 Sanofi–Aventis, Warsaw, Poland; 3 Department of Metabolic Diseases, Jagiellonian University Medical College, 15 Kopernika Street, 31–501 Kraków, Poland; 4 University Hospital, Kraków, Poland

**Keywords:** Type 2 diabetes mellitus, Insulin therapy, Insulin glargine, Long-acting insulin analog

## Abstract

**Aims:**

A variety of basal insulin preparations are used to treat patients with type 2 diabetes mellitus (T2DM). We aimed to summarize scientific evidence on relative efficacy and safety of insulin glargine (IGlar) and other insulins in T2DM.

**Methods:**

A systematic review was carried out in major medical databases up to December 2012. Relevant studies compared efficacy and safety of IGlar, added to oral drugs (OAD) or/and in combination with bolus insulin, with protamine insulin (NPH) or premixed insulin (MIX) in the same regimen, as well as with insulin detemir (IDet), in T2DM. Target HbA1c level without hypoglycemic events was considered the primary endpoint.

**Results:**

Twenty eight RCTs involving 12,669 T2DM patients followed for 12–52 weeks were included in quantitative analysis. IGlar + OAD use was associated with higher probability of reaching target HbA1c level without hypoglycemia as compared to NPH + OAD (RR = 1.32 [1.09, 1.59]) or MIX without OAD (RR = 1.61 [1.22, 2.13]) and similar effect as IDet + OAD (RR = 1.07 [0.87, 1.33]) and MIX + OAD (RR = 1.09 [0.86, 1.38]). IGlar + OAD demonstrated significantly lower risk of symptomatic hypoglycemia as compared to NPH + OAD (RR = 0.89 [0.83, 0.96]), MIX + OAD (RR = 0.75 [0.68, 0.83]) and MIX without OAD(RR = 0.75 [0.68, 0.83]), but not with IDet + OAD (RR = 0.99 [0.90, 1.08]). In basal-bolus regimens, IGlar demonstrated similar proportion of T2DM patients achieving target HbA1c as compared to NPH (RR = 1.14 [0.91, 1.44]) but higher than MIX (RR = 1.26 [1.12, 1.42) or IDet (RR = 1.38 [1.11, 1.72]). The risk of severe hypoglycemia was lower in IGlar than in NPH (RR = 0.77 [0.63, 0.94]), with no differences in comparison with MIX (RR = 0.74 [0.46, 1.20]) and IDet (RR = 1.10 [0.54, 2.25]). IGlar + OAD has comparable safety profile to NPH, with less frequent adverse events leading to treatment discontinuation than MIX + OAD (RR = 0.41 [0.22, 0.76]) and IDet + OAD (RR = 0.40 [0.24, 0.69]). Also severe adverse reactions were less common for IGlar + OAD when compared to MIX + OAD (RR = 0.71 [0.52; 0.98]).

**Conclusion:**

For the majority of examined efficacy and safety outcomes, IGlar use in T2DM patients was superior or non-inferior to the alternative insulin treatment options.

**Electronic supplementary material:**

The online version of this article (doi:10.1007/s00592-014-0698-4) contains supplementary material, which is available to authorized users.

## Introduction

Type 2 diabetes mellitus (T2DM) is a progressive disease, which requires insulin treatment when other management is no longer effective. Appropriate insulin therapy should be chosen individually to patient’s needs in order to achieve treatment goals and maintain its safety [1, 2]. In clinical practice, the flexibility of insulin titration is limited by the associated risk of hypoglycemic events, particularly when intensive insulin treatment is required [3, 4]. A growing body of evidence revealed that hypoglycemia is a predictor of poor outcome in people with T2DM, particularly it increases the risk of premature death [5–7]. Therefore, most of the clinical practice recommendations highlight that the optimal glycemic control in T2DM patients should be achieved with minimized risk of hypoglycemia [2, 8].

In general, it is recommended that at the initiation of insulin treatment in T2DM, once daily basal insulin is added to oral antidiabetic drugs (OADs) [1, 9–11]. Neutral protamine Hagedorn (NPH) has been frequently chosen as the first-line insulin; however, its use is associated with the risk of both hyper- and hypoglycemic events [12, 13]. Long-acting insulin analogs (LAAs) have been developed by modification of insulin chain in order to improve pharmacokinetic properties and decrease the risk of hypoglycemia. The first developed and most commonly prescribed LAA product is glargine (IGlar) [14]. Following the injection, IGlar forms a depot in the subcutaneous tissue, from which it is slowly absorbed. This provides a relatively uniform concentration over approximately 24 h after administration, which allows mimicking basal endogenous insulin secretion [12].

Complex pathophysiology of T2DM, its progressive nature, heterogeneous clinical picture and concomitant diseases require a variety of therapeutic options, including plural insulin regimens in order to maintain appropriate glycemic control and treatment safety. IGlar is frequently used as once daily regimen in addition to OAD. Interestingly, it has been demonstrated that early basal insulin initiation with IGlar improves FPG control and beta-cell function when compared to prolonged continuation of solely oral therapy [15]. When necessary, prandial insulin preparations can be used to intensify treatment. Therefore, IGlar is suitable for a spectrum of treatment intensities and can be used in T2DM patients at different age and various stages requiring insulin.

So far, several attempts have been undertaken to provide reliable summary of data comparing IGlar with other therapeutic options in T2DM patients [16–34]. However, available systematic reviews have assessed only selected insulin preparations and do not provide a broad clinical picture or comprehensive answer, whether IGlar use is associated with additional clinical benefits to a wide spectrum of T2DM patients. Here, for the first time, we performed a systematic review combining all data from randomized clinical trials (RCTs) in T2DM to compare efficacy and safety outcomes of IGlar with several other insulin regimens in order to make synthetic and reliable conclusions.

## Methods

### Search strategy

Following the preferred reporting items for systematic reviews and meta-analyses (PRISMA) guidelines, clinical evidence was identified through a systematic search of major databases of medical information, including Medline (via PubMed), EMBASE and the Cochrane Central Register of Controlled Trials [35]. The search strategy was constructed by combining search terms with appropriate Boolean operators in order to describe records including key words referring to both diabetes mellitus and IGlar. Databases were searched until December, 2012. Clinical trials registers (clinicaltrials.gov, ISRCTN.org) and abstracts presented at international meetings organized by the associations active in the field of diabetes (ADA, EASD) were screened for the most up-to-date clinical studies. Furthermore, references of identified articles, the websites of US Food and Drug Administration (FDA), the European Medicines Agency (EMA), Medicines and Healthcare Products Regulatory Agency (MHRA) were screened in order to retrieve potentially relevant data.

### Inclusion and exclusion criteria

Studies enrolling adults with T2DM were included. Full-text publications were considered eligible when reported RCTs directly comparing IGlar, added to OAD or/and in combination with bolus insulin, with human insulin (NPH) or insulin detemir (IDet) in the same regimens, as well as with premixed insulin (MIX). Relevant trials had to have parallel design with at least 12 weeks of follow-up; however, results of interest from the first period of cross-over studies were also accepted.

Studies enrolling a mixed population of patients with both T1DM and T2DM were excluded unless they presented separate data for the subset of individuals with T2DM. Trials recruiting only patients with non-caucasian ethnicity were not considered as race may potentially influence the effects of insulin therapy [36].

### Study selection and credibility assessment

Two analysts worked independently to select relevant studies at each stage of selection process, starting from screening of abstracts and titles and ending on thorough analysis of full texts together with credibility assessment. Discrepancies between analysts were solved by consensus. Credibility of included trials was assessed according to the scale proposed by Jadad et al., which granted from 0 to 5 points according to the presence and accuracy of methods for randomization and double blinding, and accuracy of information regarding patients lost to follow-up. Higher number of granted points reflected higher credibility of a clinical trial [37].

### Outcome of interest

The primary endpoint was glycemic control defined as a composite of target HbA1c level of ≤7 % (53 mmol/mol) without hypoglycemia. Due to differences in definitions of outcomes assessed in respective studies, the composite endpoint in this analysis encompassed either overall, severe or nocturnal hypoglycemic events; nevertheless, only homogenous results were allowed for statistical accumulation.

Key secondary endpoints in efficacy analysis included glycemic control, expressed either as the absolute reduction in mean value of glycemic parameters or percentage of patients achieving target values of HbA1c of ≤7 % (53 mmol/mol). Treatment satisfaction and quality of life were also assessed. The risk of hypoglycemic episodes as well as mean weight gain during treatment was analyzed separately. Furthermore, safety analysis was conducted, which assessed the number of patients with at least one adverse event, serious adverse event and number of subjects who prematurely withdrew from the study due to safety reason.

### Statistical analysis

Dichotomous effect measures were presented as relative risk (RR), while continuous endpoints were assessed with weighted mean difference (WMD). All estimates were presented together with 95 % confidence intervals. Between-study heterogeneity was examined using the Cochran Q test and the *I* ^2^ statistics and was considered significant when either *p* < 0.1 or *I* ^2^ ≥ 50 %. When homogeneity was confirmed, dichotomous and continuous variables were accumulated using fixed effects model with Mantel–Haenszel or inverse-variance methods, respectively. In case of statistically significant heterogeneity, DerSimonian and Laird random effect model was performed both for continuous and for dichotomous outcomes [38]. Significance of the overall effect was tested with Z-test assuming *p* < 0.05 as the level of significance. The results were processed using Sophie version 1.5.0 (meta-analysis software by HTA Consulting—verified and producing consistent results with STATA version 10.0).

## Results

**Fig. 1:**
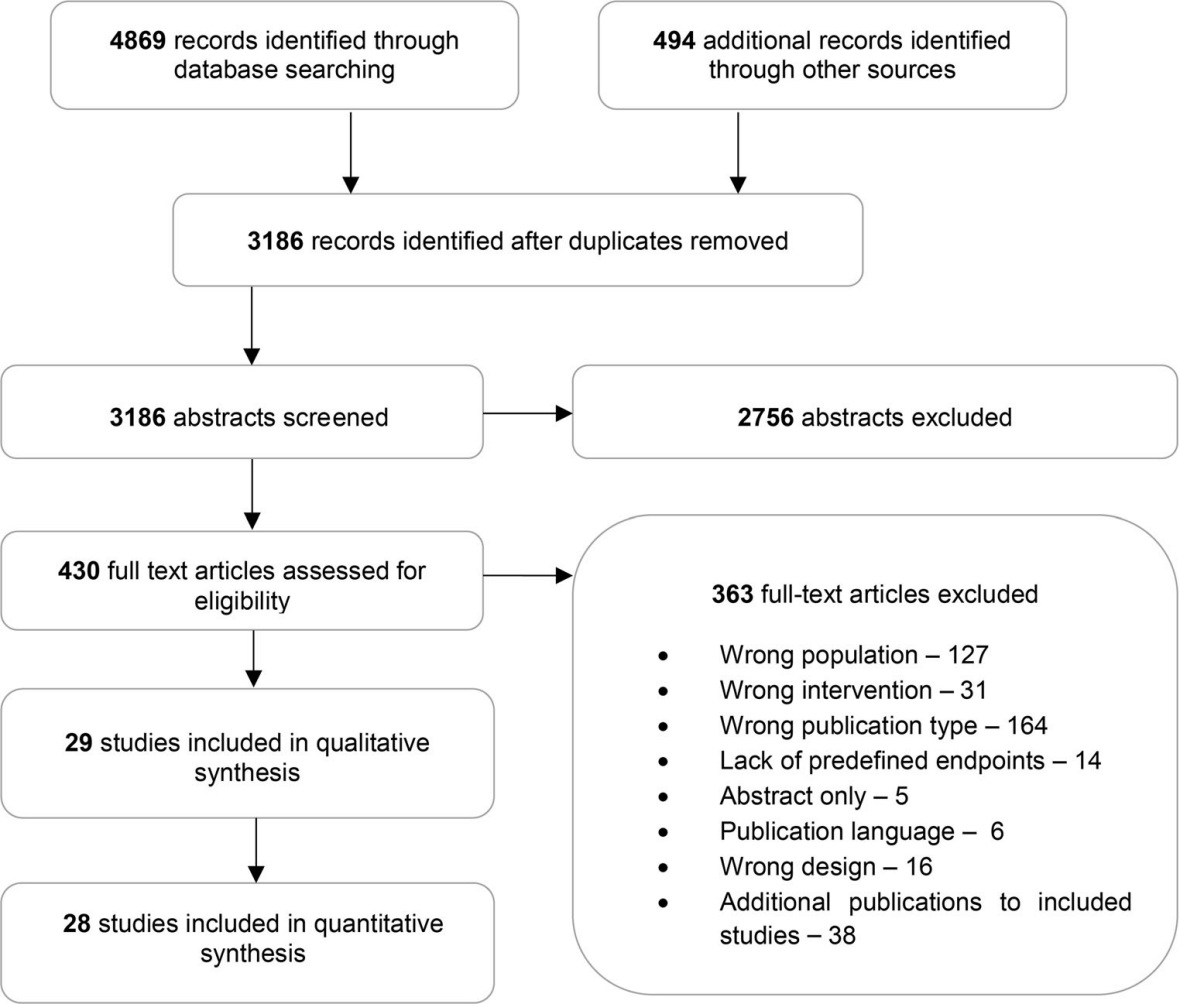
PRISMA diagram for study selection process

A total number of 3,186 records without duplicates were identified in the systematic search of medical databases. After the screening of titles and abstracts, 430 studies were considered potentially relevant and were subjected for further assessment based on full-text publications. A total number of 363 studies were subsequently excluded from the analysis due to reasons presented in PRISMA diagram (Fig. 1). Finally, 29 RCTs were included in qualitative and 28 in quantitative analysis. Overall, studies included in quantitative analysis enrolled 12,669 T2DM patients, who were followed for 12–52 weeks (Tables 1, 2).

**Table 1:** Study quality and risk of bias assessment

Study	Sponsor	Method of randomization	Double blinding	Description of lost to follow-up	Allocation concealment	Type of analysis	Total Jadad score
IGlar + OAD versus NPH + OAD							
Hsia [39]	NIH	No description	Open labeled	Sufficient	Unclear	ITT; PP	2/5
Forst [40]	Sanofi-Aventis	No description	Open labeled	Sufficient	Unclear	PP	2/5
Esposito [48]	Second University of Naples	Adequate	Open labeled	Sufficient	Adequate	PP	3/5
Eliaschewitz [41]	Sanofi-Aventis	No description	Open labeled	Sufficient	Unclear	mITT	2/5
Fritsche [42]	Sanofi-Aventis	Adequate	Open labeled	Sufficient	Unclear	mITT	3/5
Yki-Järvinen [43]	Sanofi-Aventis	Adequate	Open labeled	Sufficient	Unclear	mITT	3/5
Riddle [44]	Sanofi-Aventis	Adequate	Open labeled	Sufficient	Adequate	mITT	3/5
Massi-Bendetti [45]	Grant from Hoechst Marion Russel Deutschland Clinical Development	Adequate	Open labeled	Sufficient	Adequate	mITT	3/5
Strojek [49]	Eli Lilly	No description	Open labeled	Sufficient	Unclear	mITT	2/5
IGlar + bolus ± OAD versus NPH + bolus ± OAD							
Rosenstock [47]	Sanofi-Aventis	No description	Open labeled	Sufficient	Unclear	ITT	2/5
Koivisto [46]	Eli Lilly	No description	Open labeled	Sufficient	Unclear	mITT and PP/mITT	2/5
IGlar + OAD ± bolus versus NPH + OAD ± bolus							
Rosenstock [51]	Sanofi-Aventis	Adequate	Open labeled	Sufficient	Adequate	mITT; PP	3/5
IGlar + bolus ± OAD versus IDet + bolus ± OAD							
Hollander [67]	Novo Nordisk	Adequate	Open labeled	Sufficient	Adequate	mITT	3/5
Raskin [68]	Novo Nordisk	No description	Open labeled	Insufficient	Unclear	mITT	1/5
IGlar + OAD versus IDet + OAD							
Rosenstock [65]	Novo Nordisk	Adequate	Open labeled	Sufficient	Adequate	ITT or mITT/ITT	3/5
Swinnen [66]	Sanofi-Aventis	Adequate	Open labeled	Sufficient	Adequate	mITT, PP	3/5
IGlar + OAD versus MIX							
Al-Shaikh [52]	n/a	No description	Open labeled	Sufficient	Unclear	ITT	2/5
Janka [53]	Sanofi-Aventis	Adequate	Open labeled	Sufficient	Adequate	mITT	3/5
Schiel [54]	Sanofi-Aventis	Adequate	Open labeled	Sufficient	Adequate	mITT	3/5
IGlar + OAD versus MIX +OAD							
DURABLE 1 [55]	Eli Lilly	Adequate	Open labeled	Sufficient	Adequate	mITT/mITT	3/5
Kann [56]	Novo Nordisk	Properly described	Open labeled	Insufficient	Unclear	mITT	1/5
Raskin [57]	Novo Nordisk	Properly described	Open labeled	Sufficient	Unclear	mITT	2/5
Robbins [58]	n/a	Adequate	Open labeled	Sufficient	Adequate	mITT	3/5
Strojek [59]	Novo Nordisk	Adequate	Open labeled	Sufficient	Adequate	mITTi PP	3/5
IGlar + bolus ± OAD versus MIX ± OAD							
Bowering [60]	Eli Lilly	No description	Open labeled	Sufficient	Unclear	PP and mITT/mITT	2/5
DURABLE 2 [61]	Eli Lilly	Adequate	Open labeled	Sufficient	Adequate	mITT/mITT	3/5
GINGER [62]	Sanofi-Aventis	Adequate	Open labeled	Sufficient	Adequate	mITT/mITT	3/5
Jain [63]	Eli Lilly	Adequate	Open labeled	Sufficient	Adequate	ITT	3/5
Rosenstock [64]	Eli Lilly	Adequate	Open labeled	Sufficient	Adequate	PP/ITT	3/5

**Table 2:** Population characteristics of studies included in the analysis

Study	Study location	No. of patients (*N*)	Male gender [*n* (%)]	Mean age (years)	Duration of diabetes (years)	BMI (kg/m^2^)	HbA1c (%) (mmol/mol)	No. of daily doses	Study duration (weeks)
IGlar + OAD versus NPH + OAD									
Hsia [39]	Los Angeles	55 vs. 30	28 (50.9) vs. 21 (70.0)	51.5 vs. 53.2	9.2 vs. 7.8	31.4 vs. 32.1	9.2 vs. 7.8 (77 vs. 62)	1× vs. 1×	24
Forst [40]	Germany	14 vs. 14	2 (14.3) vs. 10 (71.4)	66.9 vs. 58.0	11.6 vs. 8.6	30.0 vs. 31.5	7.1 vs. 7.1 (54 vs. 54)	1× vs. 1×	12
Esposito [48]	Multicenter	55 vs. 55	29 (52.7) vs. 28 (50.9)	54.9 vs. 53.8	8.2 vs. 7.8	29.4 vs. 29.7	8.7 vs. 8.8 (72 vs. 73)	1× vs. 1×	36
Eliaschewitz [41]	Multicenter	231 vs. 250	99 (42.9) vs. 95 (38.0)	56.1 vs. 57.1	10.3 vs. 10.8	27.3 vs. 27.2	9.1 vs. 9.2 (76 vs. 77)	1× vs. 1×	24
Fritsche [42]	Multicenter	463 vs. 232	154 (33.3) vs. 119 (51.3)	60.5 vs. 62.0	8.6 vs. 9.3	28.7 vs. 28.9	9.1 vs. 9.1 (76 vs. 76)	1× vs. 1×	24
Yki-Järvinen [43]	Multicenter	61 vs. 49	38 (62.3) vs. 32 (65.3)	56.0 vs. 56.0	9.0 vs. 9.0	31.3 vs. 32.0	9.5 vs. 9.6 (80 vs. 81)	1× vs. 1×	36
Riddle [44]	Multicenter	367 vs. 389	202 (55.0) vs. 218 (56.0)	55.0 vs. 56.0	8.4 vs. 9.0	32.5 vs. 32.2	8.6 vs. 8.6 (70 vs. 70)	1× vs. 1×	24
Massi-Bendetti [45]	Multicenter	289 vs. 281	154 (53.3) vs. 152 (54.1)	59.6 vs. 59.4	10.2 vs. 10.5	29.3 vs. 28.8	9.0 vs. 8.9 (75 vs. 74)	1× vs. 1×	52
Strojek [49]	Multicenter	229 vs. 229	113 (49.3) vs. 122 (53.3)	57.3 vs. 58.0	9.9 vs. 9.8	31.6 vs. 30.7	8.7 vs. 8.7 (72 vs. 72)	1× vs. 1–2×	24
IGlar + bolus ± OAD versus NPH + bolus ± OAD									
Rosenstock [47]	Multicenter	259 vs. 259	150 (57.9) vs. 161 (62.2)	59.5 vs. 59.2	13.4 vs. 14.1	30.7 vs. 30.4	8.6 vs. 8.5 (70 vs. 69)	1× vs. 1–2×	28
Koivisto [46]	Multicenter	187 vs. 187	86 (46.0) vs. 76 (40.6)	60.3 vs. 59.3	11.0 vs. 11.0	33.3 vs. 33.0	8.8 vs. 8.8 (73 vs. 73)	1× vs. 1×	24
IGlar + OAD ± bolus versus NPH + OAD ± bolus									
Rosenstock [51]	Multicenter	513 vs. 504	278 (54.2) vs. 270 (53.6)	54.9 vs. 55.3	10.7 vs. 10.8	34.5 vs. 34.1	8.4 vs. 8.3 (68 vs. 67)	1× vs. 2×	260
IGlar + bolus ± OAD versus IDet + bolus ± OAD									
Hollander [67]	Multicenter	105 vs. 214	55 (52.4) vs. 130 (60.7)	58.0 vs. 59.0	13.4 vs. 13.6	31.7 vs. 31.5	8.8 vs. 8.6 (73 vs. 70)	1× vs. 1–2×	52
Raskin [68]	Multicenter	131 vs. 254	79 (60.3) vs. 131 (51.6)	55.9 vs. 55.8	11.9 vs. 12.5	33.0 vs. 32.6	8.4 vs. 8.4 (68 vs. 68)	1× vs. 1–2×	26
IGlar + OAD versus IDet + OAD									
Rosenstock [65]	Multicenter	291 vs. 291	171 (58.8) vs. 166 (57.0)	59.4 vs. 58.4	9.1 vs. 9.1	30.5 vs. 30.6	8.6 vs. 8.6 (70 vs. 70)	1× vs. 1–2×	52
Swinnen [66]	Multicenter	478 vs. 486	274 (57.3) vs. 253 (52.1)	58.7 vs. 58.0	10.1 vs. 9.7	29.7 vs. 30.6	8.7 vs. 8.7 (72 vs. 72)	1× vs. 2×	24
IGlar + OAD versus MIX									
Al-Shaikh [52]	Saudia Arabia	111 vs. 110	124 (53.7)	56.3	n/a	n/a	11.4 vs. 11.2 (101 vs. 99)	1× vs. 2×	24
Janka [53]	Multicenter	177 vs. 187	108 (61.0) vs. 107 (57.2)	60.9 vs. 60.4	9.9 vs. 9.9	29.5 vs. 29.6	8.9 vs. 8.8 (74 vs. 73)	1× vs. 2×	24
Schiel [54]	Germany	35 vs. 17	18 (51.4) vs. 9 (52.9)	63.6 vs. 69.8	14.7 vs. 16.3	31.7 vs. 30.9	8.2 vs. 8.1 (66 vs. 65)	1× vs. 2×	16
IGlar + OAD versus MIX +OAD									
DURABLE 1 [55]	Multicenter	1,046 vs. 1,045	552 (52.8) vs. 552 (52.8)	57.0 vs. 57.0	9.3 vs. 9.7	32 vs. 32	9.0 vs. 9.1 (75 vs. 76)	1× vs. 2×	24
Kann [56]	Multicenter	127 vs. 128	62 (48.8) vs. 69 (53.9)	61 vs. 61.5	10.2 vs. 10.3	30.6 vs. 29.9	8.9 vs. 9.21 (74 vs. 77)	1× vs. 2×	26
Raskin [57]	Multicenter	116 vs. 117	65 (56.0) vs. 62 (53.0)	52.3 vs. 52.6	8.3 vs. 9.5	31.4 vs. 31.5	9.8 vs. 9.7 (84 vs. 83)	1× vs. 2×	28
Robbins [58]	Multicenter	158 vs. 157	78 (49.4) vs. 79 (50.3)	58.1 vs. 57.4	12.5 vs. 11.3	32 vs. 32.1	7.8 vs. 7.8 (62 vs. 62)	1× vs. 3×	24
Strojek [59]	Multicenter	238 vs. 231	98 (41.2) vs. 108 (46.8)	56.1 vs. 55.9	9.5 vs. 9.1	29.2 vs. 29.0	8.5 vs. 8.5 (69 vs. 69)	1× vs. 1×	26
IGlar + bolus ± OAD versus MIX ± OAD									
Bowering [60]	Multicenter	212 vs. 211	105 (49,5) vs. 93 (44,1)	56.3 vs. 56.7	10.0 vs. 10.6	27.5 vs. 27.9	9.0 vs. 9.0 (75 vs. 75)	1–3× vs. 1–3×	48
DURABLE 2 [61]	Multicenter	370 vs. 374	187 (50.5) vs. 199 (53.2)	56.5 vs. 55.7	9.4 vs. 9.4	32.7 vs. 32.8	8.0 vs. 8.0 (64 vs. 64)	1× vs. 2–3×	24
GINGER [62]	Multicenter	153 vs. 157	83 (54.2) vs. 75 (47.8)	60.2 vs. 60.9	12.8 vs. 12.5	30.3 vs. 29.8	8.6 vs. 8.5 (70 vs. 69)	1× vs. 2×	58
Jain [63]	Multicenter	195 vs. 188	101 (51.8) vs. 86 (45.7)	59.9 v*s*. 58.9	12.0 vs. 11.4	28.8 vs. 29.1	9.3 vs. 9.5 (78 vs. 80)	1–3× vs. 1–3×	36
Rosenstock [64]	Multicenter	187 vs. 187	98 (52.4) vs. 99 (52.9)	54.0 vs. 55.4	11.2 vs. 10.9	34.8 vs. 34.1	8.89 vs. 8.83 (74 vs. 73)	1× vs. 3×	24

Nine studies compared IGlar versus NPH [39–47], while in two others, patients from the comparatory group received neutral protaminated insulin lispro (NPL) [48, 49]. However, all these studies were analyzed together as NPL demonstrates similar pharmacokinetic and pharmacodynamic properties to NPH [50]. In nine studies, protamine insulin was administered once daily, while in the remaining two trials, patients were allowed to receive protaminated insulin according to either *qd* or *bid* schemes [47, 49]. Nine studies assessed basal + OAD regimen; in four of which patients received one oral drug either metformin [40, 43] or a sulphonylurea derivative [41, 42], while in the remaining five RCTs participants could be treated with more than one OAD [39, 44, 45, 48, 49]. In two studies assessing basal + bolus regimen, either human or lispro insulin was used as prandial insulin and OAD therapy was allowed but not obligatory [46, 47]. Additionally, one long-term RCT comparing IGlar + OAD with NPH + OAD was identified. However, this study was not included in quantitative analysis due to heterogeneous treatment and much longer follow-up (260 weeks) when compared to the remaining RCTs [51].

Thirteen studies assessed IGlar in comparison with MIX, of which three compared IGlar + OAD with MIX monotherapy [52–54], while in eight of them patients received insulins in combination with OAD in both groups [55–59]. Remaining 5 RCTs assessed IGlar + bolus ± OAD regimen in comparison with MIX ± OAD [60–64]. Premixed insulin analogs were used as comparators in most RCTs, except for participants of trials comparing IGlar + OAD with mix monotherapy [52–54] and 59 % of subjects from another study assessing basal + bolus regimen in comparison with MIX [62], who were treated with human premixed insulins.

Finally, four studies assessed IGlar in comparison with IDet. Both LAA were administered in basal + OAD regiment in two RCTs [65, 66] and according to basal + bolus scheme in the remaining two [67, 68].

In most studies, the follow-up was not longer than 6 months, while three studies reported the outcomes after around 1 year of treatment (Table 2) [45, 60, 62]. The credibility of included RCTs, assessed according to the Jadad scale, oscillated between 1 and 3 points on the 5-point scale and was mainly downgraded due to the lack of double blinding (Table 1).

### IGlar versus NPH

#### Glycemic control

The meta-analysis of 2 RCTs assessing basal + OAD regimen demonstrated a favorable effect of IGlar over NPH with respect to target HbA1c without nocturnal hypoglycemia (RR = 1.32 [1.09, 1.59]), while the mean reduction in HbA1c level was comparable in both arms (9 RCTs; WMD = −0.03 % [−0.10, 0.04] (−0.3 mmol/mol [−1.1; 0.4])). No difference between IGlar and NPH, both in combination with prandial insulin, was observed with respect to the mean reduction of HbA1c (2 RCTs; WMD = 0.02 % [−0.30, 0.35] (0.2 mmol/mol [−3.3; 3.8])) as well as the number of T2DM patients achieving target HbA1c (1 RCT; RB = 1.14 [0.91; 1.44]) [46].

#### Hypoglycemia

Meta-analysis of five studies assessing IGlar in comparison with NPH, both added to OAD, revealed a borderline difference toward lower risk of overall hypoglycemia in patients treated with IGlar (RR = 0.92 [0.84, 1.001]). Moreover, IGlar + OAD significantly reduced number of patients experiencing symptomatic (6 RCTs; RR = 0.89 [0.83, 0.96]) and nocturnal events (6 RCTs; RR = 0.63 [0.51; 0.77]). The risk of severe hypoglycemia was comparable between interventions (5 RCTs; RR = 0.76 [0.47, 1.23]) [41, 42, 44, 45, 49].

Meta-analysis of the 2 RCTs assessing basal + bolus scheme demonstrated less frequent nocturnal hypoglycemic events in patients treated with IGlar as compared to protamine insulin (RR = 0.77 [0.63, 0.94]). Additionally, a tendency toward lower risk of severe hypoglycemic events was shown in patients treated with IGlar (RR = 0.22 [0.05, 1.02]) [46, 47].

#### Weight gain

IGlar and NPH did not differ significantly with respect to weight gain when administered within basal + OAD (6 RCTs; WMD = 0.36 kg [−0.12, 0.84]) or basal + bolus ± OAD regimens (2 RCTs; WMD = −0.45 kg [−1.52, 0.61]).

#### Treatment satisfaction and quality of life

One RCT reported superior treatment satisfaction of IGlar over NPH, both added to OAD (WMD = 0.60 [0.07; 1.13]) [41].

#### Safety

No difference between interventions was demonstrated with regard to the risk of adverse events related to study drug and the risk of study discontinuations due to adverse events. The incidence of serious adverse events (SAE) was generally low and did not reveal any difference between IGlar and NPH in either treatment regimens. Similarly, both basal insulins were associated with similar risk of overall adverse events when administered according to basal + OAD (RR = 1.00 [0.93, 1.09]) or basal + bolus ± OAD (1 RCT; RR = 1.13 [0.88, 1.44]) regimens, respectively. Only single cases of mortality were reported in two RCTs comparing IGlar + OAD with NPH + OAD [42, 45], and in one RCT assessing both basal insulins in basal + OAD scheme with no differences between treatment arms [46].

### IGlar versus premixed insulins (MIX)

#### Glycemic control

Single RCT reported that significantly more patients treated with IGlar + OAD achieved target HbA1c without nocturnal hypoglycemia when compared to MIX monotherapy (RR = 1.61 [1.22, 2.13]) [53]. Additionally, IGlar combined with OADs exerted a greater reduction in mean level of HbA1c (3 RCTs; WMD = −0.36 % [−0.54, −0.18] (−3.9 mmol/mol [−5.9; −2.0])) and was associated with a higher chance of reaching target HbA1c (2 RCTs; RR = 1.49 [1.03, 2.16]).

A single study demonstrated that both insulin preparations added to OAD have comparable efficacy with respect to primary endpoint defined as achievement of target HbA1c < 7 % (53 mmol/mol) without either overall (RR = 0.97 [0.67, 1.40]) or nocturnal hypoglycemic events (RR = 1.09 [0.86, 1.38]) [59]. However, MIX + OAD provided larger reduction of HbA1c (5 RCTs; WMD = 0.26 % [0.12, 0.40] (2.8 mmol/mol [1.3, 4.4])) and allowed to achieve target HbA1c in a higher number of patients (5 RCTs; RR = 0.79 [0.66, 0.94]).

Meta-analysis of five studies demonstrated that IGlar added to prandial insulin compared with MIX ± OAD showed a trend toward lower mean HbA1c (WMD −0.19 % [−0.43,0.06] (−2.1 mmol/mol [−4.7, 0.7])) and was associated with a higher percentage of patients who reached target HbA1c (RR = 1.26 [1.12, 1.42]).

#### Hypoglycemia

A meta-analysis of two studies comparing IGlar + OAD versus MIX monotherapy demonstrated no difference between groups with respect to the risk of overall hypoglycemia (RR = 0.90 [0.78; 1.04]) [53, 54]. However, Janka et al. [53] demonstrated significantly lower number of symptomatic (2.62 vs. 5.73 events/patient-year; *p* < 0.001) as well as nocturnal (0.051 vs. 1.04 events/patient-year; *p* < 0.05) hypoglycemic events in IGlar group. Severe hypoglycemia was uncommon in both arms [53, 54].

IGlar as compared to MIX, both administered together with OAD, demonstrated lower risk of overall (3 RCTs; RR = 0.88 [0.82, 0.95]) and symptomatic hypoglycemia (3 RCTs; RR = 0.75 [0.68, 0.83]), while no differences were found with respect to the risk of nocturnal (2 RCTs; RR = 1.01 [0.90, 1.14]) and severe events (5 RCTs; RR = 0.86 [0.30, 4.43]) [55, 57–59].

IGlar added to prandial insulin when compared to MIX ± OAD therapy demonstrated similar impact with respect to all assessed hypoglycemic endpoints including overall (2 RCTs; RR = 1.01 [0.93; 1.10]) [62, 63], symptomatic (2 RCTs; RR = 1.02 [0.95; 1.10]) [62, 64], severe (5 RCTs; RR = 0.74 [0.46, 1.20]) [60–64] and nocturnal events (3 RCTs; RR = 0.98 [0.87; 1.10]) [62–64].

#### Weight gain

Meta-analysis of three RCTs comparing IGlar added to OAD with MIX monotherapy demonstrated comparable weight gain in both groups (WMD = −2.02 kg [−5.11; 1.07]), although this result has limited credibility due to a significant between-study heterogeneity (*p* = 0.03) [52–54].

Pooled estimate of three studies showed lower mean body weight gain in patients receiving IGlar + OAD than in those who were on MIX + OAD therapy (WMD = −1.27 kg [−1.56, −0.97]) [55, 57, 58]. On the other hand, IGlar combined with prandial insulin provided comparable effect on weight gain as MIX ± OAD (5 RCTs; WMD = 0.37 kg [−0.20; 0,94]).

#### Treatment satisfaction and quality of life

Irrespectively of assessed treatment scheme, no evidence was found for the difference in overall treatment satisfaction or quality of life between IGlar and MIX [54, 55, 60]. However, in one study IGlar + OAD provided within-group improvement in hypoglycemic, cardiovascular and psychological/cognitive subdomains of DSC-R, while patients treated with MIX + OAD did not report significant difference from baseline [55].

#### Safety

The proportion of premature withdrawals due to adverse events was lower in IGlar + OAD group when compared to MIX + OAD (5 RCTs; RR = 0.41 [0.22, 0.76]), but not to MIX monotherapy (2 RCTs; RR = 0.52 [0.13, 1.99]). Comparable number of withdrawals due to adverse events was observed for the comparison between IGlar + bolus ± OAD vs. MIX ± OAD (4 RCTs; RR = 1.44 [0.63, 3.28]). In comparison with MIX + OAD, IGlar decreased the number of severe adverse events when used with OAD (3 RCTs; RR = 0.71 [0.52, 0.98]), but not as adjunctive to prandial insulin (5 RCTs; RR = 1.05 [0.78, 1.42]). Single cases of mortality were reported in 2 RCTs comparing IGlar with MIX, both added to OAD, and in 4 RCTs comparing IGlar + bolus ± OAD versus MIX ± OAD with no difference between treatment arms. No evidence for the difference between IGlar and MIX with respect to both overall adverse events and treatment associated adverse events was found regardless of treatment schemes that were directly compared.

### IGlar versus IDet

#### Glycemic control

Two RCTs reported no difference between IGlar and IDet, both added to OAD, with respect to proportion of patients reaching target HbA1c level with either no overall (RR = 1.05 [0.83, 1.35]) or symptomatic hypoglycemic events (RR = 1.07 [0.87, 1.33]), respectively [65, 66]. Meta-analysis of both RCTs demonstrated comparable reduction in mean HbA1c in both groups (WMD = 0.05 % [−0.07, 0.16] (0.5 mmol/mol [−0.8, 1.7])) [65, 66]. The proportion of patients, who reached a target point of HbA1c ≤ 7 % (53 mmol/mol), was similar between treatment arms (2 RCTs; RR = 0.95 [0.86, 1.06]) [65, 66].

Results of single RCTs assessing basal + bolus ± OAD regimen revealed superiority of IGlar over IDet with respect to the primary endpoint defined as HbA1c reduction below 7 % (53 mmol/mol) with no evidence for overall hypoglycemia (RR = 1.41 [1.12, 1.78]), but no difference between interventions was found with respect to the number of patients achieving target HbA1c without symptomatic hypoglycemia (RR = 1.21 [0.75, 1.95]) [67, 68]. IGlar was associated with a larger reduction in mean HbA1c level (2 RCTs; WMD = −0.25 % [−0.40, −0.09] (−2.7 mmol/mol [−4.4; 1.0])) and allowed to reach a target HbA1c level (<7 % (53 mmol/mol)) by significantly more patients when compared to IDet (2 RCTs; RR = 1.23 [1.03, 1.47]) [67, 68].

#### Hypoglycemia

The risk of hypoglycemia in patients treated with both LAA added to OAD was comparable with respect to overall (1 RCT, RR = 1.05 [0.93, 1.19]), symptomatic (2 RCTs; RR = 0.99 [0.90, 1.08]), severe (2 RCTs; RR = 1.31 [0.70, 2.45]) and nocturnal hypoglycemic events (1 RCT; RR = 0.98 [0.77, 1.24]).

Both LAA administered according to basal + bolus ± OAD regimen were associated with comparable risk of overall, symptomatic, severe and nocturnal hypoglycemic episodes.

#### Weight gain

Meta-analysis of two RCTs comparing IGlar versus IDet, both added to OAD therapy, demonstrated higher body weight gain in IGlar group (WMD = 0.77 kg [0.44, 1.11]) [65, 66]. Similarly, IGlar was also associated with a higher body weight increase as compared to IDet, when both analogs were administered together with prandial insulins (2 RCTs; WMD = 1.24 kg [0.59, 1.89]) [67, 68].

#### Treatment satisfaction and quality of life

One study comparing both LAA in basal + OAD regimen reported that IGlar was associated with a higher treatment satisfaction when compared to IDet as measured with DTSQ questionnaire (for overall result *p* < 0.001), but no difference was shown when measured with DSC-R, WHO-5 Well Being and HFS questionnaires [66].

#### Safety

The number of premature withdrawals due to adverse events was significantly lower in IGlar group as compared to IDet, when both interventions were administered in addition to OAD therapy (RR = 0.40 [0.24, 0.69]), but not as adjuncts to bolus insulin (RR = 0.54 [0.22; 1,32]). The risk of serious adverse events did not differ between both LAA administered either together with OAD (1 RCT; RR = 1.26 [0.87, 1.83]) or in combination with prandial insulin (2 RCTs; RR = 0.71 [0.43, 1.16]). Similarly, meta-analysis of 2 RCTs demonstrated a comparable risk between IGlar and IDet in basal + bolus regimens with respect to overall adverse events (RR = 1.02 [0.94, 1.21]) [67, 68]. Pooled results from two studies comparing both interventions added to OAD treatment revealed four times lower risk of application site reactions in IGlar group as compared to IDet (RR = 0.22 [0.07; 0.55]) [65, 66]. Only one death was reported, in patient receiving IDet + OAD [65].

## Discussion

Pharmacotherapy of T2DM usually starts from monotherapy with metformin, and it is further intensified by adding OADs of other classes or injectable GLP-1 agonists; nevertheless, many patients will eventually require insulin, usually beginning from one injection of basal insulin preparation [1, 2, 69]. Many insulin products have been developed so far to cover the full spectrum of T2DM patients’ needs. IGlar has favorable pharmacokinetic and pharmacodynamics properties that allow providing constant insulin activity over 24 h with only a single injection, and is the most widely prescribed LAA [12]. Since numerous systematic reviews have been published in order to combine the outcomes of many RCTs comparing IGlar with various comparators, the additional value of our meta-analysis will be hereby discussed [16–34, 70]. Firstly, majority o earlier meta-analyses and secondary studies did not provide a full picture of clinical efficacy and safety of IGlar, since they were focused exclusively on certain aspects of insulin therapy (for example, intensive insulin treatment) [16, 17, 31]. Secondly, the other studies did not take into account the complexity of insulin treatment and pooled together trials recruiting patients with heterogenous clinical characteristics, subjected to different treatment models or accumulated results for different insulin preparations, e.g., insulin glargine with insulin detemir and premixed insulin analogs with human biphasic insulins [16, 19, 23, 25, 26, 29, 31, 33, 70]. Finally, several reviews did not attempt to accumulate included studies and presented only qualitative assessment, which significantly limited accuracy and precision of the conclusions [21, 24, 26, 28, 30, 32, 34].

In order to address the heterogeneity of T2DM and describe the efficacy and safety of investigated interventions in the context of different treatment regimens, we performed two separate analyses for each insulin preparation used for comparison. The first one involved less intensified insulin therapy (e.g., IGlar ± OAD), while the second one concerned intensive insulin therapy (IGlar + bolus). Therefore, our analysis is comprehensive and allows us to draw very accurate and reliable conclusions. Additionally, we identified several recent primary studies, which allowed us to receive more up-to-date results and perform more thorough investigation of heterogeneity than it was previously reported.

The primary efficacy outcome was defined as the percentage of patients achieving target HbA1c level without hypoglycemia and encompassed either overall severe, nocturnal or symptomatic hypoglycemic events, as the definitions of outcomes varied between respective studies. Although the heterogeneous reporting of composite outcomes limited between-trial comparability, still the combination of glycemic control and hypoglycemia serves as the most representative measure of treatment effectiveness. Indeed, it is well known that a decrease in the HbA1c level is usually achieved at the cost of higher risk of hypoglycemic episodes. These episodes are associated with increased mortality and decreased quality of life as shown in different cohorts T2DM patients [5–7, 71]. Thus, reaching glycemic target without hypoglycemic events seems to bring particular benefits. The results of our comparative analysis indicate that IGlar is an option with favorable efficacy and acceptable safety profile. IGlar + OAD increased the proportion of patients reaching target HbA1c level without hypoglycemic events as compared to NPH + OAD. Although available evidence did not allow us to compare IGlar + bolus with NPH + bolus, the analysis of individual endpoints demonstrated comparable reduction of HbA1c in each arm, but with concomitantly lower rate of symptomatic and nocturnal hypoglycemia in IGlar group. These results are consistent with most of the available systematic reviews comparing IGlar and NPH, which reported similar effect on HbA1c level with concomitantly lower risk of hypoglycemia for IGlar, particularly in terms of nocturnal events [18, 20, 21, 28].

Available systematic reviews showed that MIX, as compared to LAA, was associated with better glycemic control but also with a higher incidence of hypoglycemia [23, 33]. However, neither of these analyses took an effort to interpret the results in the context of relatively high degree of between-study heterogeneity with respect to treatment in the control groups. Indeed, in available RCTs patients enrolled to control groups received either monotherapy with human premixed insulins or a combination of biphasic analog insulins with OAD therapy. In the current analysis, we have shown that the superior effect of MIX + OAD over IGlar with respect to mean HbA1c decrease was associated with greater weight gain, and higher risk of symptomatic hypoglycemia. We observed an advantage of IGlar over human premixed insulins (without OAD) with respect to both the reduction of HbA1c and the incidence of hypoglycemia, which was not shown in previous reviews.

Finally, we also demonstrated a favorable effect of IGlar over IDet in basal + bolus regimen, as IGlar use was associated with a higher percentage of patients reaching target HbA1c without the experience of hypoglycemia. When considering basal + OAD therapy, both insulins exerted similar effect on the primary endpoint. These observations differ from those presented by other authors, which can be explained by several limitations of previous reports that have been resolved in the current analysis. In 2008, Fakhoury et al. [17] reported an advantage of IDet over IGlar in relation to the risk of hypoglycemia, with comparable metabolic control as measured by HbA1c. However, these results were derived from an indirect comparison including evidence published before March 2007; therefore, have much less credibility than current meta-analyses performed on most up-to-date head-to-head comparisons. Another systematic review by Swinnen et al. [70] found no differences between IDet and IGlar, both in terms of glycemic control and the risk of hypoglycemia. However, the credibility of those results was limited by a high degree of statistical heterogeneity, which probably reflected between-study differences in treatment regimens. Within this analysis, we separately assessed both basal + OAD and basal + bolus regimens, which was reasonable as both schemes are usually used for different disease severities. These separate analyses allowed to remove statistical heterogeneity and to obtain more precise results indicating the advantage of IGlar + bolus over IDet + bolus with respect to glycemic control.

The current review has some potential limitations. First, our meta-analysis could be criticized for choosing a fixed HbA1c target below 7.0 % as a component of the composite primary endpoint, while the individualization is an important element of the contemporary diabetes management. Nevertheless, most major clinical guidelines maintained HbA1c level of <7.0 % as a general treatment goal, which can be advantageous for majority of patients with diabetes [72–74]. The treatment target may be relieved mainly in patients with long-lasting diabetes, short life expectancy, existing comorbidities, etc. Since most studies included in our meta-analysis excluded individuals for whom less stringent glycemic control target could be applied, such as elderly individuals with severe chronic complications, the definition of the primary endpoint adopted in this paper is justified. Additionally, due to the short duration of studies included in the current meta-analysis and low incidence of malignancies, we were not able to analyze between-treatment differences in the risk of malignancies. Such analysis would have been of potential clinical importance taking into account earlier studies searching for potential association between T2DM and pharmacological therapies used for its treatment and the risk of cancer [75–81].

As depicted on a study flow diagram, 363 papers did not meet inclusion criteria for the analysis due to various reasons. This may raise concerns whether the data selection process was correct. Such a high number of publications excluded from the meta-analysis are to some extent a consequence of a highly sensitive strategy used for database searching, which allowed us to retrieve all important studies. This also, however, resulted in a relatively large number of less relevant data including reviews, letters, conference proceedings and others, which had to be removed in further steps of selection process.

Finally, the quality of included studies, which were mainly designed as open-labeled comparisons, should be discussed. Of note, proper maintenance of glycemic control requires continuous insulin titration, which could not be performed when patients or physicians are unaware of the assigned treatment since various insulins require different dosing. Blinding to treatment allocation would lead to suboptimal glucose levels control with excessive rate of hypoglycemia, and therefore, open-labeled design is justified in studies assessing insulin therapy.

Finally, yet another limitation of this review is a short follow-up in the majority of included RCTs. Most studies had a duration up to 6 months, which may not provide a fully reliable picture of relative efficacy of insulin treatment in long-term perspective. The only longitudinal RCT identified within our systematic review was designed to compare IGlar versus NPH, both added to OAD therapy [51]. Nevertheless, due to the long follow-up, the protocol allowed for modification of both OAD therapy and insulin treatment, so that prandial insulins could be introduced or withdrawn during the study. Indeed, during the mean follow-up of 260 weeks, most of the patients in both treatment groups received human prandial insulins. The study demonstrated a difference in HbA1c reduction in favor of twice daily NPH insulin, which was most likely due to higher insulin dose and higher percentage of patients who were co-administered with prandial insulin in NPH group. Indeed, a post hoc analysis for patients treated solely with basal insulins and OAD demonstrated nearly equivalent reduction of HbA1c in both groups [51]. Additionally, IGlar was associated with fewer patients experiencing severe hypoglycemia and with no apparent difference in mean body gain [51].

In conclusion, for the majority of examined efficacy and safety outcomes, IGlar use in T2DM patients was superior or at least non-inferior to the alternative insulin treatment options (Table 3).

**Table 3:** Summary of the results for the comparison between IGlar and other insulin preparations

Outcome	IGlar vs. NPH		IGlar vs. MIX			IGlar vs. IDet	
	+OAD	+bolus ± OAD	IGlar: +OAD MIX: MT	+OAD	IGlar: +bolus ± OAD MIX: +OAD	+OAD	+bolus ± OAD
Target HbA1c without hypoglycemia							
Overall	n/a	n/a	n/a	No difference	n/a	No difference	FavoursIGlar
Nocturnal	FavoursIGlar	n/a	FavoursIGlar	No difference	n/a	n/a	n/a
Symptomatic	n/a	n/a	n/a	n/a	n/a	No difference	No difference
HbA1c reduction	No difference	No difference	FavoursIGlar	Favours MIX	No difference	No difference	FavoursIGlar
Target HbA1c	No difference	No difference	FavoursIGlar	Favours MIX	FavoursIGlar	No difference	FavoursIGlar
FPG reduction	FavoursIGlar	No difference	FavoursIGlar	No difference	No difference	FavoursIGlar	No difference
Target FPG level	No difference	No difference	FavoursIGlar	FavoursIGlar	No difference	FavoursIGlar	No difference
Body weight gain	No difference	No difference	No difference	FavoursIGlar	No difference	FavoursIDet	FavoursIDet
Treatment satisfaction (DTSQ)	FavoursIGlar	n/a	No difference	n/a	n/a	FavoursIGlar	n/a
Quality of life (DSC-R)	n/a	n/a	n/a	FavoursIGlar	No difference	FavoursIGlar	n/a
Overall hypoglycemia	No difference	No difference	FavoursIGlar	FavoursIGlar	No difference	No difference	No difference
Symptomatic hypoglycemia	FavoursIGlar	No difference	FavoursIGlar	FavoursIGlar	No difference	No difference	No difference
Severe hypoglycemia	No difference	No difference	No difference	No difference	No difference	No difference	No difference
Nocturnal hypoglycemia	FavoursIGlar	FavoursIGlar	FavoursIGlar	No difference	No difference	No difference	No difference
Overall AEs	No difference	No difference	No difference	No difference	No difference	n/a	No difference
AEs associated with treatment	No difference	No difference	No difference	No difference	FavoursIGlar	No difference	n/a
AEs: injection site reactions	No difference	No difference	n/a	n/a	n/a	FavoursIGlar	n/a
Withdrawals due to AEs	No difference	No difference	No difference	FavoursIGlar	No difference	FavoursIGlar	No difference
SAEs	No difference	No difference	n/a	FavoursIGlar	No difference	No difference	No difference

## Electronic supplementary material

Below is the link to the electronic supplementary material.

Supplementary material 1 (DOCX 59 kb)

Supplementary material 2 (DOCX 43 kb)
