# Supplementary material for: Systematic review and meta-analysis of randomized clinical trials comparing efficacy and safety outcomes of insulin glargine with NPH insulin, premixed insulin preparations or with insulin detemir in type 2 diabetes mellitus
Source: Acta Diabetol. 2015 Jan 14;52(4):649–62. doi: 10.1007/s00592-014-0698-4 (PMC4506471; doi:10.1007/s00592-014-0698-4)
Supplement: Supplementary file 1 — Supplementary material 1 (DOCX 59 kb) [file 592_2014_698_MOESM1_ESM.docx]

Table 1.
Dichotomous outcomes for comparison between IGlar and other insulin preparations

| Endpoint | No of studies | Follow-up [week] | IGlar | Comparator | RR [95% CI] | Test for significance  (p value) | Test for heterogeneity | |
| --- | --- | --- | --- | --- | --- | --- | --- | --- |
|  |  |  | n/N (%) | n/N (%) |  |  | (p value) | *I^2^* |
| IGlar+OAD vs. NPH+OAD | | | | | | | | |
| Target HbA1c without nocturnal hypoglycemia | 2 | 24 | 179/594 (30) | 145/636 (23) | 1.32 [1.09, 1.59] | 0.004 | 0.36 | 0.0% |
| Target HbA1c level (≤7.0%) | 4 | 24-36 | 358/709 (50) | 367/705 (52) | 0.99 [0.90, 1.09] | 0.831 | 0.86 | 0.0% |
| Target FPG/FBG level (≤ 5.6 mmol/L) | 3 | 24-36 | 253/656 (39) | 255/697 (37) | 1.05 [0.92, 1.21] | 0.450 | 1.00 | 0.0% |
| Overall hypoglycemia | 5 | 12-52 | 496/882 (56) | 354/631 (56) | 0.92 [0.84, 1.00] | 0.054 | 0.69 | 0.0% |
| Symptomatic hypoglycemia | 6 | 24-52 | 686/1322 (52) | 623/1077 (58) | 0.89 [0.83, 0.96] | 0.001 | 0.30 | 18.2% |
| Severe hypoglycemia | 5 | 24-52 | 31/1579 (2) | 36/1381 (3) | 0.76 [0.47, 1.23] | 0.264 | 0.26 | 23.5% |
| Nocturnal hypoglycemia | 6 | 24-52 | 283/1322 (21) | 378/1077 (35) | 0.63 [0.51, 0.77] | <0.001 | 0.07 | 51% |
| Discontinuations due to AEs | 5 | 24-52 | 23/1586 (1) | 21/1387 (2) | 0.88 [0.50, 1.55] | 0.654 | 0.69 | 0.0% |
| Treatment-associated AEs | 2 | 24-52 | 55/520 (11) | 52/531 (10) | 1.10 [0.77, 1.57] | 0.590 | 0.12 | 58.9% |
| Serious AEs | 5 | 12-52 | 23/585 (4) | 19/592 (3) | 1.22 [0.68, 2.19] | 0.510 | 0.33 | 13.2% |
| Overall AEs | 6 | 12-52 | 491/874 (56) | 489/873 (56) | 1.00 [0.93, 1.09] | 0.929 | 0.67 | 0.0% |
| Injection site reactions | 2 | 24–52 | 26/520 (5) | 26/531 (5) | 1.05 [0.62, 1.77] | 0.864 | 0.43 | 0.0% |
| Mortality | 2 | 24-52 | 3/752 (<1) | 7/513 (1) | 0.31 [0.07, 1.35] | 0.120 | 0.26 | 21.9% |
| IGlar+bolus±OAD vs. NPH+bolus±OAD | | | | | | | | |
| Target HbA1c level (≤7.0%) | 1 | 24 | 86/180 | 75/179 | 1.14 [0.91, 1.44] | 0.264 | - | - |
| Target FPG/FBG level (≤ 6.7 mmol/L) | 1 | 28 | 77/259 (30) | 70/259 (27) | 1.10 [0.84, 1.45] | 0.496 | - | - |
| Overall hypoglycemia | 1 | 24 | 119/187 (64) | 105/187 (56) | 1.13 [0.96, 1.34] | 0.141 | - | - |
| Symptomatic hypoglycemia | 1 | 28 | 159/259 (61) | 173/259 (67) | 0.92 [0.81, 1.05] | 0.201 | - | - |
| Severe hypoglycemia | 2 | 24-28 | 2/446 (1) | 9/446 (2) | 0.22 [0.05, 1.02] | 0.054 | 0.66 | 0.0% |
| Nocturnal hypoglycemia | 2 | 24-28 | 117/446 | 152/446 | 0.77 [0.63, 0.94] | 0.0104 | 0.87 | 0.0% |
| Discontinuations due to AEs | 2 | 24-28 | 9/446 (2) | 8/446 (2) | 1.12 [0.45, 2.78] | 0.8112 | 0.43 | 0.0% |
| Treatment-associated AEs | 2 | 24-28 | 27/446 (6) | 23/446 (5) | 1.17 [0.69, 1.98] | 0.559 | 0.14 | 54.6% |
| Serious AEs | 1 | 24 | 9/187 (5) | 14/187 (8) | 0.64 [0.29, 1.45] | 0.287 | - | - |
| Overall AEs | 1 | 24 | 81/187 (43) | 72/187 (39) | 1.13 [0.88, 1.44] | 0.348 | - | - |
| Iniection site reactions | 1 | 24 | 0/187 (0) | 1/187 (1) | 0.33 [0.01, 8.13] | 0.500 | - | - |
| Mortality | 1 | 24 | 0/187 (0) | 1/187 (1) | 3.00 [0.12, 73.17] | 0.500 | - | - |
| IGlar + OAD vs MIX monotherapy | | | | | | | | |
| Target HbA1c without nocturnal hypoglycemia | 1 | 24 | 81/177 (46) | 53/187 (28) | 1.61 [1.22, 2.13] | <0.001 | - | - |
| Target HbA1c level (≤7.0%) | 2 | 24 | 150/288 (52) | 107/297 (36) | 1.49 [1.03, 2.16] | 0.032 | 0.06 | 71.0% |
| Target FPG/FBG level (≤5.5 mmol/l) | 2 | 24 | 102/288 (35) | 48/297 (16) | 2.18 [1.61, 2.95] | <0.001 | 0.81 | 0.0% |
| Overall hypoglycemia | 2 | 16-24 | 132/212 (62) | 140/204 (69) | 0.90 [0.78, 1.04] | 0.146 | 0.78 | 0.0% |
| Discontinuations due to AEs | 2 | 16-24 | 4/212 (2) | 6/204 (3) | 0.52 [0.13, 1.99] | 0.338 | 0.10 | 62.5% |
| Treatment-associated AEs | 1 | 24 | 8/177 (5) | 10/187 (5) | 0.85 [0.34, 2.09] | 0.716 | - |  |
| Overall AEs | 1 | 24 | 89/177 (50) | 92/187 (49) | 1.02 [0.83, 1.26] | 0.836 | - | - |
| IGlar+OAD vs MIX+OAD | | | | | | | | |
| Target HbA1c without hypoglycemia | 1 | 26 | 45/232 (19) | 45/225 (20) | 0.97 [0.67, 1.40] | 0.871 | - | - |
| Target HbA1c without nocturnal hypoglycemia | 1 | 26 | 92/323 (40) | 82/225 (36) | 1.09 [0.86, 1.38] | 0.479 | - | - |
| Target HbA1c level (≤7.0%) | 5 | 24-28 | 665/1679 (40) | 802/1672 (48) | 0.79 [0.66, 0.94] | <0.001 | 0.01 | 69.4% |
| Target FPG/FBG level (≤6.7 mmol/l) | 1 | 24 | 91/158 (58) | 37/157 (24) | 2.44 [1.79, 3.34] | <0.001 | - | - |
| Overall hypoglycemia | 3 | 24-26 | 680/1442 (47) | 764/1433 (53) | 0.88 [0.82, 0.95] | <0.001 | 0.21 | 35.7% |
| Symptomatic hypoglycemia | 3 | 24-26 | 413/1411 (29) | 548/1404 (39) | 0.75 [0.68, 0.83] | <0.001 | 0.79 | 0.0% |
| Severe hypoglycemia | 5 | 24-28 | 6/639 (<1) | 7/633 (1) | 0.86 [0.30, 4.43] | 0.773 | 0.85 | 0.0% |
| Nocturnal hypoglycemia | 2 | 24 | 370/1204 (31) | 365/1202 (30) | 1.01 [0.90, 1.14] | 0.838 | 0.76 | 0.0% |
| Discontinuations due to AEs | 5 | 24-28 | 14/1679 (1) | 34/1672 (2) | 0.41 [0.22, 0.76] | 0.005 | 0.82 | 0.0% |
| Treatment-associated AEs | 1 | 24 | 4/158 (3) | 7/157 (4) | 0.57 [0.17, 1.90] | 0.359 | - | - |
| Serious AEs | 3 | 24-26 | 61/1331 (5) | 86/1330 (6) | 0.71 [0.52, 0.98] | 0.035 | 0.39 | 0.0% |
| Overall AEs | 3 | 24-26 | 272/517 (53) | 294/510 (58) | 0.91 [0.82, 1.02] | 0.106 | 0.10 | 56.3% |
| Mortality | 2 | 24-26 | 2/1287 (<1) | 5/1284 (<1) | 0.45 [0.10, 2.02] | 0.299 | 0.17 | 47.1% |
| IGlar+bolus±OAD vs MIX+OAD | | | | | | | | |
| Target HbA1c level (≤7.0%) | 5 | 24-52 | 397/1076 (37) | 316/1080 (29) | 1.26 [1.12, 1.42] | <0.001 | 0.26 | 24.7% |
| Target FPG/FBG level (≤5.5 mmol/l) | 1 | 52 | 39/153 (25) | 26/157 (17) | 1.54 [0.99, 2.40] | 0.057 | - | - |
| Overall hypoglycemia | 2 | 36-52 | 295/393 (75) | 294/396 (74) | 1.01 [0.93, 1.10] | 0.790 | 0.77 | 0.0% |
| Symptomatic hypoglycemia | 2 | 24-52 | 272/340 (80) | 270/344 (78) | 1.02 [0.95, 1.10] | 0.647 | 0.22 | 33.1% |
| Nocturnal hypoglycemia | 3 | 24-52 | 282/580 (48) | 289/583 (50) | 0.98 [0.87, 1.10] | 0.731 | 0.79 | 0.0% |
| Severe hypoglycemia | 5 | 24-52 | 27/1162 (2) | 37/1168 (3) | 0.74 [0.46, 1.20] | 0.219 | 0.65 | 0.0% |
| Discontinuations due to AEs | 4 | 24-52 | 13/952 (1) | 9/960 (1) | 1.44 [0.63, 3.28] | 0.383 | 0.62 | 0.0% |
| Treatment-associated AEs | 1 | 48 | 0/212 (0) | 7/211 (3) | 0.07 [0.00, 1.15] | 0.063^a^ | - | - |
| Serious AEs | 5 | 24-52 | 78/1164 (7) | 75/1171 (6) | 1.05 [0.78, 1.42] | 0.750 | 0.60 | 0.0% |
| Overall AEs | 3 | 24-52 | 302/607 (50) | 294/610 (48) | 1.03 [0.92, 1.16] | 0.565 | 0.72 | 0.0% |
| Mortality | 4 | 24-52 | 5/977 (<1) | 3/984 (<1) | 1.45 [0.44, 4.82] | 0.542 | 0.39 | 0.1% |
| IGlar+OAD vs. IDet+OAD | | | | | | | | |
| Target HbA1c without hypoglycemia | 1 | 52 | 90/259 (35) | 82/248 (33) | 1.05 [0.83, 1.35] | 0.689 | - | - |
| Target HbA1c without symptomatic hypoglycemia | 1 | 24 | 131/478 (27) | 124/486 (26) | 1.07 [0.87, 1.33] | 0.506 | - | - |
| Target HbA1c level (≤7.0%) | 2 | 24-52 | 346/737 (47) | 361/734 (49) | 0.95 [0.86, 1.06] | 0.373 | 0.47 | 0.0% |
| Target FPG/FBG level (≤ 6 mmol/l) | 1 | 52 | 150/259 (58) | 114/248 (46) | 1.26 [1.06, 1.49] | 0.008 | - | - |
| Overall hypoglycemia | 1 | 52 | 191/291 (66) | 182/291 (63) | 1.05 [0.93, 1.19] | 0.437 | - | - |
| Symptomatic hypoglycemia | 2 | 24-52 | 400/769 (52) | 410/777 (53) | 0.99 [0.90.1.08] | 0.778 | 0.82 | 0.0% |
| Nocturnal hypoglycemia | 1 | 52 | 93/291 (32) | 95/291 (33) | 0.98 [0.77, 1.24] | 0.859 | - | - |
| Severe hypoglycemia | 2 | 24-52 | 22/769 (3) | 17/777 (2) | 1.31 [0.70, 2.45] | 0.399 | 0.66 | 0.0% |
| Discontinuations due to AEs | 2 | 24-52 | 18/769 (3) | 45/777 (6) | 0.40 [0.24, 0.69] | <0.001 | 0.48 | 0.0% |
| Treatment-associated AEs | 1 | 52 | 32/291 (11) | 40/291 (14) | 0.80 [0.52, 1.24] | 0.315 | - | - |
| Serious AEs | 1 | 52 | 53/291 (18) | 42/291 (14) | 1.26 [0.87, 1.83] | 0.219 | - | - |
| Injection site reactions | 2 | 24–52 | 4/769 (1) | 22/777 (3) | 0.20 [0.07, 0.55] | 0.002 | 0.24 | 28.6% |
| Mortality | 1 | 52 | 0/291 (0) | 1/291 (<1) | 0.33 [0.01, 8.15] | 0.500 | - | - |
| IGlar+bolus±OAD vs. IDet+bolus±OAD | | | | | | | | |
| Target HbA1c without hypoglycemia | 1 | 26 | 67/128 (52) | 93/251 (37) | 1.41 [1.12, 1.78] | 0.003 | - | - |
| Target HbA1c without symptomatic hypoglycemia | 1 | 52 | 22/105 (21) | 37/214 (17) | 1.21 [0.75, 1.95] | 0.426 | - | - |
| Target HbA1c level (≤7.0%) | 2 | 26-52 | 108/233 (46) | 175/465 (38) | 1.23 [1.03, 1.47] | 0.025 | 0.13 | 56.4% |
| Target level FPG/FBG level (≤6.0 mmol/l) | 1 | 52 | 96/105 (91) | 185/214 (86) | 1.06 [0.98, 1.14] | 0.166 | - | -- |
| Overall hypoglycemia | 2 | 26-52 | 182/236 (77) | 353/470 (75) | 1.03 [0.94, 1.12] | 0.550 | 0.27 | 19.3% |
| Symptomatic hypoglycemia | 1 | 52 | 68/105 (65) | 130/214 (61) | 1.07 [0.89, 1.27] | 0.480 | - | - |
| Nocturnal hypoglycemia | 2 | 26-52 | 109/236 (46) | 214/470 (46) | 1.01 [0.86, 1.20] | 0.865 | 0.263 | 20.3% |
| Severe hypoglycemia | 2 | 26-52 | 11/236 (5) | 20/470 (4) | 1.10 [0.54, 2.25] | 0.798 | 0.760 | 0.0% |
| Discontinuations due to AEs | 2 | 26-52 | 6/238 (3) | 22/472 (5) | 0.54 [0.22, 1.32] | 0.177 | 0.87 | 0.0% |
| Serious AEs | 2 | 26-52 | 19/236 (8) | 54/470 (11) | 0.71 [0.43, 1.16] | 0.168 | 0.17 | 47.3% |
| Overall AEs | 2 | 26-52 | 181/236 (77) | 354/470 (75) | 1.02 [0.94, 1.21] | 0.639 | 0.21 | 36.0% |

1. The difference was significant when assessed with relative difference: RD = -0.03 [-0.06, -0.01]
