# Supplementary material for: Systematic review and meta-analysis of randomized clinical trials comparing efficacy and safety outcomes of insulin glargine with NPH insulin, premixed insulin preparations or with insulin detemir in type 2 diabetes mellitus
Source: Acta Diabetol. 2015 Jan 14;52(4):649–62. doi: 10.1007/s00592-014-0698-4 (PMC4506471; doi:10.1007/s00592-014-0698-4)
Supplement: Supplementary file 2 — Supplementary material 2 (DOCX 43 kb) [file 592_2014_698_MOESM2_ESM.docx]

Table 2.
Continuous outcomes for comparison between IGlar and other insulin preparations

| Endpoint | | No of studies | Follow-up [week] | IGlar | Comparator | MD [95% CI] | Test for significance  (p value) | Test for heterogeneity | |
| --- | --- | --- | --- | --- | --- | --- | --- | --- | --- |
|  |  |  |  | n/N (%) | N (%) |  |  | (p value) | *I^2^* |
| IGlar+OAD vs. NPH+OAD | | | | | | | | | |
| HbA1c reduction [%] | | 9 | 12-52 | 1764 | 1528 | -0.03 [-0.10, 0.04] | 0.339 | 0.39 | 4.9% |
| FPG/FBG reduction [mmol/l] | | 8 | 12-52 | 1535 | 1300 | -0.14 [-0.27, -0.01] | 0.034 | 0.68 | 0.0% |
| Weight gain [kg] | | 6 | 24-52 | 1464 | 1210 | 0.36 [-0.12, 0.84] | 0.140 | 0.031 | 59.3% |
| Treatment satisfaction [DTSQ] | | 1 | 24 tyg. | 231 | 250 | 0.60 [0.07, 1.13] | 0.0262 | - | - |
| IGlar+bolus±OAD vs. NPH+bolus±OAD | | | | | | | | | |
| HbA1c reduction [%] | | 2 | 24-28 | 439 | 438 | 0.02 [-0.30, 0.35] | 0.886 | 0.025 | 80.2% |
| Weight gain [kg] | | 2 | 24-28 | 439 | 438 | -0.45 [-1.52, 0.61] | 0.405 | 0.037 | 77.1% |
| IGlar + OAD vs MIX monotherapy | | | | | | | | | |
| HbA1c reduction [%] | | 3 | 16-24 | 323 | 314 | -0.36 [-0.54, -0.18] | <0.001 | 0.12 | 53.7% |
| FPG/FBG reduction [mmol/l] | | 2 | 16-24 | 212 | 204 | -0.93 [-1.39, -0.46] | <0.001 | 0.43 | 0.0% |
| Weight gain [kg] | | 2 | 24 | 288 | 297 | -2.02 [-5.11, 1.07] | 0.199 | 0.03 | 79.6% |
| Treatment satisfaction [DTSQ] | | 1 | 16 | 35 | 17 | 1.21 [-2.37, 4.79] | 0.507 | - | - |
| IGlar+OAD vs MIX+OAD | | | | | | | | | |
| HbA1c reduction [%] | 5 | | 24-28 | 1679 | 1672 | 0.26 [0.12, 0.40] | <0.001 | 0.02 | 65.3% |
| FPG/FBG reduction [mmol/l] | 4 | | 24-28 | 1447 | 1436 | -0.54 [-1.28, 0.19] | 0.147 | <0.01 | 90.9% |
| Weight gain [kg] | 3 | | 24-28 | 1320 | 1319 | -1.27 [-1.56, -0.97] | <0.001 | 0.16 | 44.8% |
| IGlar+bolus±OAD vs MIX+OAD | | | | | | | | | |
| HbA1c reduction [%] | 5 | | 24-52 | 1110 | 1114 | -0.19 [-0.43, 0.06] | 0.143 | 0.01 | 68.9% |
| Weight gain [kg] | 5 | | 24-52 | 905 | 906 | 0.37 [-0.20, 0.94] | 0.202 | 0.07 | 53.6% |
| IGlar+OAD vs. IDet+OAD | | | | | | | | | |
| HbA1c reduction [%] | 2 | | 24-52 | 753 | 754 | 0.05 [-0.07, 0.16] | 0.442 | 0.37 | 0.0% |
| FPG/FBG reduction [mmol/l] | 2 | | 24-52 | 750 | 754 | -0.30 [-0.58, -0.02] | 0.034 | 0.59 | 0.0% |
| Weight gain [kg] | 2 | | 24-52 | 769 | 777 | 0.77 [0.44, 1.11] | <0.001 | 0.80 | 0.0% |
| IGlar+bolus±OAD vs. IDet+bolus±OAD | | | | | | | | | |
| HbA1c reduction [%] | 2 | | 26-52 | 233 | 465 | -0.25 [-0.40, -0.09] | 0.002 | 0.42 | 0.0% |
| FPG/FBG reduction [mmol/l] | 2 | | 26-52 | 233 | 464 | -0.12 [-0.54, 0.30] | 0.564 | 0.18 | 0.1% |
| Weight gain [kg] | 2 | | 26-52 | 236 | 468 | 1.24 [0.59, 1.89] | <0.001 | 0.63 | 0.0% |
